# A Clash of Authority in Quarantine Matters

**Published:** 1900-04

**Authors:** 


### TEXAS MEDICAL JOURNAL.

AUSTIN, TEXAS.

EDITED AND PUBLISHED BY

F. E. DANIEL, M. D., AND S. E. HUDSON, M. D.

ESTABLISHED JULY, 1885.

PUBLISHED MONTHLY.—SUBSCRIPTION $1.00 A YEAR.

VOL. XV. AUSTIN, APRIL, 1900. No. 10.

### A Clash of Authority in Quarantine Matters.

**"JONES PAYS THE FREIGHT."**

SMALLPOX in a mild form is prevailing extensively in Texas, many counties being infected. The cost of local quarantine falling, under the law (?), on the county, has been, in many instances, very burdensome. The taxpayers are kicking in some counties against the burden, and in Colorado county, and, we understand, Williamson county, the county commissioners have refused to institute any quarantine proceedings, refusing to go to the expense incidental thereto; and the question has been raised if it is not the duty of the State to take charge of local epidemics? There is much misunderstanding on the subject, and some of the dailies are severely rebuking the obstinate county commissioners for violating, as the *Statesman* says, "the plain letter of the law." Well, the writer of this knows something about that same law, and takes the liberty of saying that the county commissioners are exactly right in refusing to burden their constituents with quarantine of smallpox or any other disease, and that it is somebody else, higher in authority, who is "violating the plain letter of the law." Interested readers are referred to the quarantine law published in the TEXAS MEDICAL JOURNAL, October, 1899. In support of the statements made above, we quote Section 11, and part of Section 10 of said law. I know of no State law which makes it *mandatory* for counties to institute and pay for quarantine; no law imposing penalty for failure to do so. On the contrary, it seems that under the State law quarantine is exclusively the function of the Governor or State Health Officer. Section 11 reads:

"Whenever on the coast of Texas or *elsewhere in this State* the authorities of any county, town or city, fail, refuse, or neglect to establish quarantine, as provided in the preceding article [Section.—Ed.], then and in that event the Governor shall have the power and it SHALL BE HIS DUTY to appoint a health officer and to prescribe such regulations for the government of the same as he may deem necessary."

Section 10 (referred to) provides that "all cost and expense of enforcing and maintaining the general quarantine, OR SUCH AS ARE ORDERED BY THE GOVERNOR OR STATE HEALTH OFFICER shall be paid out of the fund appropriated for quarantine purposes. * * * Those (officers) commissioned by the Governor to guard against threatened epidemics, or those *temporarily assigned to duty by the Health Officer of the State* under the provisions of Section 4 of this act, shall be allowed and paid not more than $5 per day and such other pay for extra expenses actually incurred as may be deemed just by the Governor and State Health Officer (paid by the State, of course).

It is true Section 15, the wording of which is ambiguous, authorizes county commissioners to "direct" (it does not say "shall" or "may") the county physician to declare and maintain quarantine, etc., to provide everything needed, and says the "county *shall assume and pay all lawful expenses incurred* by local quarantine; but suppose that the commissioners shall "fail, refuse, or neglect" to establish quarantine, where is the remedy? In that event, as just pointed out, the law makes it the duty of the Governor and State Health Officer to place a temporary health officer in charge, and pay him; and as in Section 9 it is provided that the State shall pay the expenses of detaining sick or suspected persons in quarantine, etc., it seems a fair construction of the law to say that when the State takes charge of a local epidemic where the county authorities "fail, neglect, or refuse" to act—the expense shall be paid by the State, as is the salary of the temporary officer acting for the State; and that the State has no right to fix the said cost on the county, in the event of the failure, neglect, or refusal of said county to voluntarily assume it. We should like to see the case tested in the courts.

